# Parallel Fixed Point Implementation of a Radial Basis Function Network in an FPGA

**DOI:** 10.3390/s141018223

**Published:** 2014-09-29

**Authors:** Alisson C. D. de Souza, Marcelo A. C. Fernandes

**Affiliations:** Department of Computer Engineering and Automation, Center of Technology, Federal University of Rio Grande do Norte—UFRN, Natal 59078-970, Brazil; E-Mail: alisson.camara@gmail.com

**Keywords:** artificial neural network, ANN, radial basis function, RBF, FPGA, fixed point, Simulink, system generator

## Abstract

This paper proposes a parallel fixed point radial basis function (RBF) artificial neural network (ANN), implemented in a field programmable gate array (FPGA) trained online with a least mean square (LMS) algorithm. The processing time and occupied area were analyzed for various fixed point formats. The problems of precision of the ANN response for nonlinear classification using the XOR gate and interpolation using the sine function were also analyzed in a hardware implementation. The entire project was developed using the System Generator platform (Xilinx), with a Virtex-6 xc6vcx240t-1ff1156 as the target FPGA.

## 1. Introduction

Artificial neural networks (ANNs) are computational techniques that employ mathematical models inspired by the neural structures of intelligent organisms, which acquire knowledge based on past and present experience. These intelligent organisms possess extremely complex sets of cells, called neurons; the structure of an ANN is composed of processing units called artificial neurons, whose functioning involves parallel and distributed interconnections [1]. One of the popular ANN architectures is the radial basis function (RBF) networks that employ least mean square (LMS) algorithms in their training.

From the implementation perspective, one of the main problems of RBFs relates to the lack of a methodology for the definition of their topology in terms of the number of centers, which, on the one hand, has a positive influence on their computational cost. There are several heuristic techniques available to assist the designer with network topology [1]. However, there is a consensus that the topology depends on the problem under investigation [2]. A commonly used procedure is to test various topologies for different sets of information. Nonetheless, this practice is time-consuming and can only be applied in cases of off-line training or when statistical information concerning the data space is known.

Various solutions for the implementation of application-specific integrated circuits (ASICs) have been proposed in order to accelerate the functioning and training of ANNs [3–5]. However, implementations in ASICs fix the architecture and the algorithm implemented, resulting in poor flexibility and/or high cost. Nevertheless, with advances in reconfigurable hardware structures, there has been renewed focus on the implementation of ANNs, and dedicated hardware structures are available that are flexible in terms of their topology and training algorithm. Currently, the most widely used architectures for reconfigurable hardware are the FPGAs, which can provide performance similar to an ASIC, with the advantage of rapid prototyping. There have been several studies concerning the implementation of multilayer perceptrons (MLPs) in FPGAs [2,3,6–11], the implementation of RBFs in FPGAs [12–21], as well as the implementation of SOMin FPGA [22].

This work presents a parallel hardware implementation of an RBF-type ANN. The implementation is made at a fixed point and is aimed at reconfigurable hardware architectures of the type FPGA. Different from earlier studies [2,3,6–8,12–20], here, a detailed analysis of the implementation of the RBF is provided, considering aspects, including processing time, delay time and the precision of the response. The proposed method was tested using two scenarios that have been widely reported in the literature. The first concerns the problem of nonlinear classification associated with the XOR gate, and the second considers the problem of interpolation using the sine function. All of the results were obtained using the System Generator (Xilinx) development platform [23], with a Virtex-6 xc6vcx240t-1ff1156 FPGA. The System Generator is a design tool over Simulink of MATLAB [24].

## 2. Radial Basis Function Networks

RBF networks have become increasingly popular in the domain of artificial networks [1]. The structure of an RBF network consists of multiple layers; the processing is the feedforward type, and the training can be either supervised (as used in the present work), or hybrid, where a supervised method is combined with an unsupervised method.

### 2.1. Architecture

**Figure 1.**
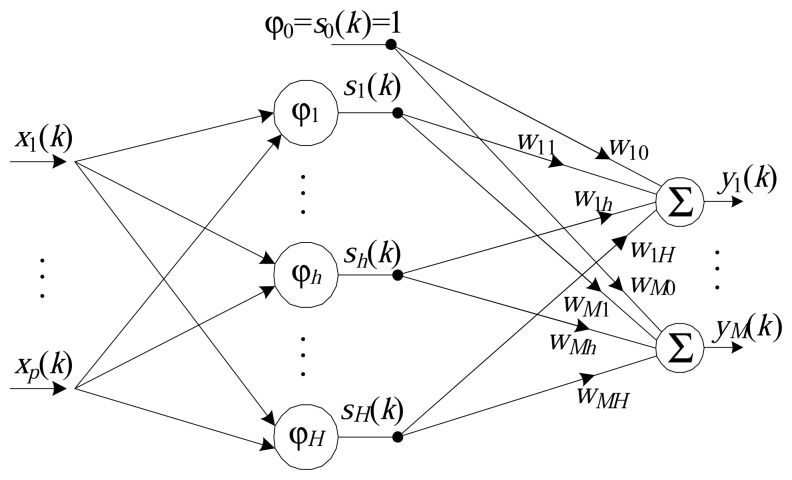
Architecture of the radial basis function (RBF).

The basic structure of an RBF network (Figure 1) consists of only three layers. The first layer is the connection of the model with the medium and is composed of *p* inputs. The second (or hidden) layer is composed of *H* radial basis functions (also known as neurons) and performs a nonlinear transformation of the input vector space into an internal vector space, whose dimensions are usually larger (*H* > *p*). The final (output) layer transforms the internal vector space into an output using a set of *M* linear neurons.

Radial basis functions only produce responses that are significantly different from zero when the input pattern is located within a small region of the domain, and each function requires a center and a scaling parameter, *β*. The most widely used radial basis function is the Gaussian function [1]. The *h*-th function, *ϕ_h_* (·), can be expressed by:

(1) $$s_{h}(k)=\varphi_{h}(\text{x}(k))=e^{\left(-\beta v_{h}(k)\right)}$$

where $\beta =\frac{1}{2\sigma^{2}}$ and *v_h_*(*k*), which represents the input distance, **x**(*k*), in relation to the *h*-th center, **c***_h_*, expressed as:

(2) $$v_{h}(k)={\left\|\text{x}(k)-{\text{c}}_{h}\right\|}^{2}$$

The vector of centers associated with the *h*-th radial function is characterized as:

(3) $${\text{c}}_{h}=\left[\begin{array}{c} c_{h,1}\\ \vdots \\ c_{h,i}\\ \vdots \\ c_{h,p} \end{array}\right]$$

and the vector of inputs as:

(4) $$\text{x}(k)=\left[\begin{array}{c} x_{1}(k)\\ \vdots \\ x_{i}(k)\\ \vdots \\ x_{p}(k) \end{array}\right]$$

Equation (2) can therefore be rewritten and expressed by:

(5) $$v_{h}(k)=\sum_{i=1}^{p}{\left(x_{i}(k)-c_{h,i}\right)}^{2}$$

The output of the *m*-th neuron of the output layer, *N_m_*, can be characterized as:

(6) $$y_{m}(k)=\sum_{h=0}^{H}w_{mh}s_{h}(k),\text{for}\;m=1,\ldots ,M$$

Substituting Equation (1) in Equation (6) gives:

(7) $$y_{m}(k)=\sum_{h=0}^{H}w_{mh}(k)e^{\left(-\frac{1}{2\sigma_{h}^{2}}{\left\|\text{x}(k)-{\text{c}}_{h}\right\|}^{2}\right)}$$

where *w_mh_* is the synaptic weight between neuron *h* of the hidden layer and neuron *m* of the output layer. At each instant *k*, the RBF network receives a vector of inputs, **x**(*k*), and generates an output vector, **y**(*k*), expressed by:

(8) $$\text{y}(k)=\left[\begin{array}{c} y_{1}(k)\\ \vdots \\ y_{m}(k)\\ \vdots \\ y_{M}(k) \end{array}\right]$$

### 2.2. Training Algorithm

Due to the difference between the hidden layer and the output layer, the training of RBF networks is usually divided into two parts. The first part concerns the nonlinear optimization performed by the hidden layer. In this, the input vector, **x**(*k*), is processed by means of functions present in the hidden layer, characterized by Equation (1). Several different strategies can be used to determine the centers, **c***_h_* [1]. Different from conventional methods, where fixed centers are selected randomly [1], the strategy employed here was to select fixed centers deterministically. The second part of the training involved calculation of the weights, *w_mh_*, between the hidden layer and the output layer. The weights can be obtained using the pseudo-inverse method [1] or the LMS algorithm [25]. The online LMS procedure used here is an iterative technique that optimizes the mean squared error (MSE) function using the gradient descent method with classical stochastic estimation (exchanging the mathematical approach in favor of an instantaneous estimate) [25]. The parameters are adjusted every instant, *k*, using the expression:

(9) $$w_{mh}(k)=w_{mh}(k-1)+\mu e_{m}(k)s_{h}(k)$$

where *μ* is the learning rate and *e_m_* (*k*) is the training error associated with the *m*-th output neuron in the *k*-th instant. The error can be expressed by:

(10) $$e_{k}(k)=d_{m}(k)-y_{m}(k)$$

where *d_m_*(*k*) is the desired value for the *m*-th output neuron. The online LMS is less computationally complex, compared to the pseudo-inverse method that requires a nonquadratic matrix inversion calculation in order to obtain the weights, *w_mh_* (*k*) [25].

## 3. Architecture and Implementation

### 3.1. General Structure

**Figure 2.**
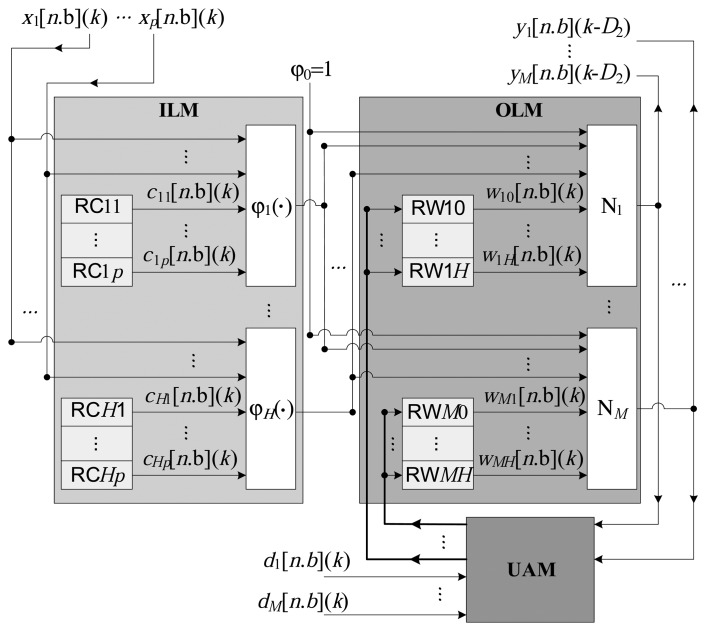
General structure of the RBF network implemented in an FPGA.

**Figure 3.**
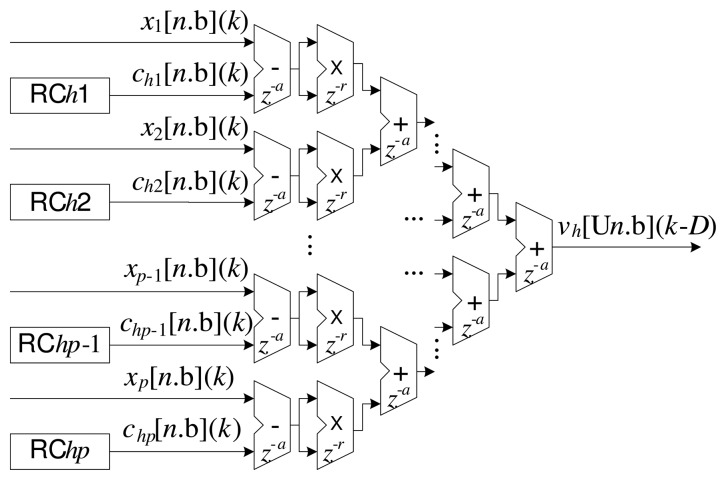
The implementation as described in Equations (2) and (5).

Figure 2 presents the general architecture of the proposed implementation. All of the variables and constants are implemented in a fixed point, utilizing a resolution of *n* bits, of which *b* bits represent the fractional part and (*n* − *b*) bits represent the integer part. The representation [*n*.*b*] is used for the fixed point variables with a sign, and [U*n*.*b*] is used for the variables without a sign. The architecture is composed of three large modules, characterized as the intermediate layer, output layer and updating algorithm, as illustrated in Figure 3.

(1) Intermediate layer module (ILM): This consists of the calculation of the radial basis functions, according to Equation (1), to generate the signal, *s_h_*(*k*), from the input, **x**(*k*). Processing of the *H* radial functions, *ϕ_h_*(·), is performed in parallel, from the centers stored in the local registers possessing *n* bits. Each center, *c_h,i_*, (see Equation (3)) is stored in the register RC*hi*, as illustrated in Figure 2.
(2) Output layer module (OLM): This represents the processing performed by the *M* output neurons, as described in Equation (6). In this module, the *M* neurons also function in parallel, generating the signal **y**(*k*) (see Equation (8)) from the input, **x**(*k*).
(3) Updating algorithm module (UAM): This module is responsible for implementing the updating algorithm, which, in the present case, is the online LMS. The updating of each synaptic weight, *w_mh_*(*k*), at the *k*-th instant, is performed in parallel, according to Equation (9). As shown in Figure 2, the weights, *w_mh_*, are stored in local registers of *n* bits, here termed RW*mh*.

The ILM, OLM and UAM modules function sequentially. For each instant *k*, the vector of inputs, **x**(*k*), is processed to generate the output vector, **y**(*k*), and all of the synaptic weights are calculated in order to update the registers, RW*mh*. It is important to note that it would be possible to further improve the performance of the RBF by using the modules in the form of a pipeline (this was not employed in the present work).

### 3.2. Radial Basis Functions

**Figure 4.**
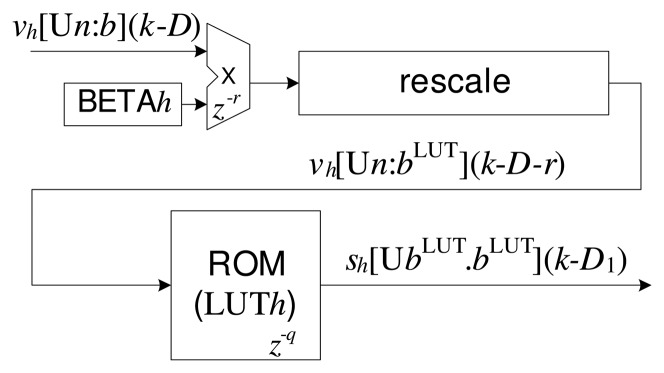
The implementation with respect to Equation (1).

Figures 3 and 4 present details of the processing associated with the *h*-th radial basis function of the ILM.

Figure 3 illustrates the implementation corresponding to Equation (5). In this case, the overall implementation is performed in a partially parallel manner, as described previously [2,7,26]. The delays associated with the additions and multiplications are also shown in Figure 3. Each addition can be implemented with a delay *z*^−^*^a^* and each multiplication with a delay *z*^−^*^r^*. The insertion of delays in the operations relaxes the conditions of routing between the cells in the FPGA, principally in complex operations, such as those involving multipliers. On the other hand, the introduction of delays slows the response of the system, which can often be disadvantageous [23]. The calculation of the total delay, *D*, with respect to Equation (5), can be expressed as:

(11) $$D=r+a+a\log_{2}(p)$$

A variety of techniques are available for the calculation of nonlinear functions in hardware, and in the case of FPGAs, one of the most common is the use of lookup tables (LUTs) in ROM memory [2,7,26]. Figure 4 shows the proposed implementation for the *h*-th radial function (see Equation (1)), in which read-only memory (ROM) was used to develop the LUT. For each *h*-th radial function, there is an LUT*h* and a multiplication operation to adjust the scaling parameter, *β* (stored in the BETA*h* register). In order to obtain greater resolution associated with each *h*-th LUT*h*, the *v_h_*(*k*) variable can be rescaled to a new format expressed by $\left[\text{U}n.b_{h}^{\text{LUT}}\right]$, in which $b_{h}^{\text{LUT}}$ is a new value for the number of bits of the fractional part, calculated using:

(12) $$b_{h}^{\text{LUT}}=n-\left\lceil \log_{2}\left(\left\lceil \delta_{h}^{\max}\right\rceil \right)\right\rceil$$

where:

(13) $$\delta_{h}^{\max}=\max\{v_{h}[n.b]\cdot \beta \}$$

Equation (13) determines the greatest distance of all of the input sets to the *h*-th center, and this information enables the number of bits of the integer part to be reduced to $\left(n-b_{h}^{\text{LUT}}\right)$, while increasing the number of bits of the fractional part in $b_{h}^{\text{LUT}}$. Figure 4 illustrates the re-scaling step performed after the multiplication operation and before the LUT step.

As *v_h_* (*k*) · *β* > 0, for any *k*, the value of the response of the radial function is limited to 0 ≤ *s_h_* (*k*) ≤ 1 and can therefore be represented at a fixed point as $\left[\text{U}b_{h}^{\text{LUT}}\cdot b_{h}^{\text{LUT}}\right]$, in which the number of bits of the integer part is zero. The values stored in the *h*-th LUT can be characterized by the vector LUT*_h_*, expressed as:

(14) $${\text{LUT}}_{h}=\left[\begin{array}{c} s_{h}^{0}\left[\text{U}b_{h}^{\text{LUT}}\cdot b_{h}^{\text{LUT}}\right]\\ \vdots \\ s_{h}^{j}\left[Ub_{h}^{\text{LUT}}\cdot b_{h}^{\text{LUT}}\right]\\ \vdots \\ s_{h}^{\left(P-1\right)}\left[Ub_{h}^{\text{LUT}}\cdot b_{h}^{\text{LUT}}\right] \end{array}\right]$$

where *P* is the depth of the LUT, characterized as:

(15) $$P=\left\lceil \left\lceil \delta_{h}^{\max}\right\rceil \cdot 2^{b_{h}^{\text{LUT}}}\right\rceil$$

and:

(16) $$s_{h}^{j}\left[\text{U}b_{h}^{\text{LUT}}\cdot b_{h}^{\text{LUT}}\right]=e^{t_{h}^{j}}$$

where:

(17) $$t_{h}^{j}=\frac{j\cdot \left\lceil \delta_{h}^{\max}\right\rceil }{P-1}\;\text{for}\;j=0,\ldots ,P-1$$

The delay associated with Equation (1), *D*_1_, can be expressed as the sum of the delay corresponding to the calculation of distance (see Equation (11)) and the delay corresponding to processing of the LUT:

(18) $$D_{1}=r+q+D$$

where *q* is the LUT delay *(z*^−^*^q^*)

### 3.3. Output Layer Neurons

**Figure 5.**
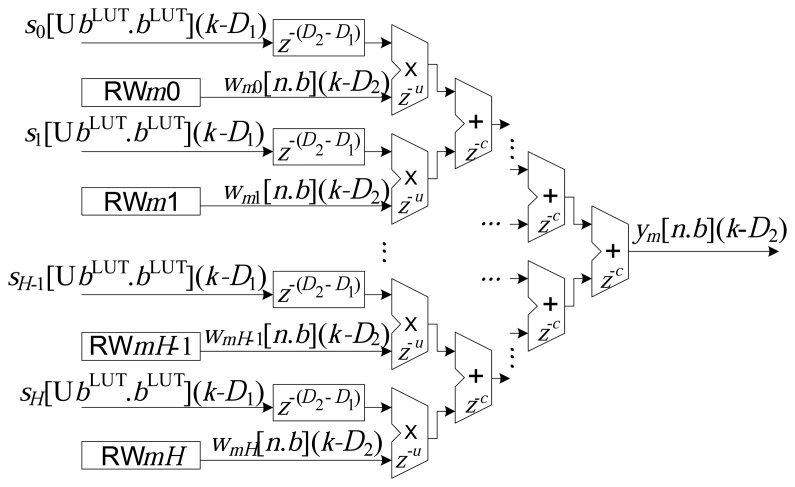
The implementation of the *m*-th neuron, *N_m_*, of the output layer (see Equation (6)).

The implementation of each *m*-th neuron, *N_m_*, of the output layer is illustrated in Figure 5. Similar to the processing presented in Figure 3, the sums of the products amongst the inputs and the synaptic weights were also implemented in a partially parallel manner [2,7,26].

In the case of the implementations associated with the output neurons, the addition operations can have delays of *z*^−^*^u^* samples, and the multiplication operations can have delays of *z*^−^*^c^* samples. The total accumulated delay, from the input to the output of the RBF, can be expressed by:

(19) $$D_{2}=D_{1}+u+c\log_{2}(H)+c$$

### 3.4. Online LMS Algorithm

**Figure 6.**
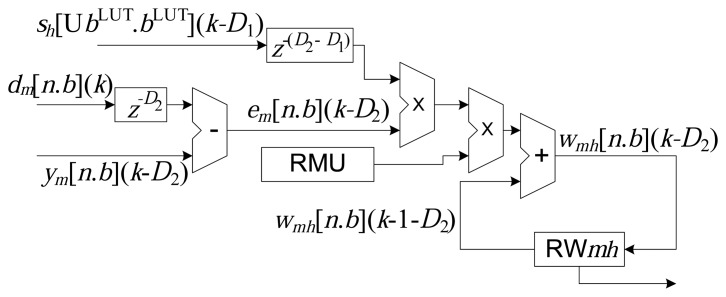
Updating of the synaptic weights, *w_mh_* (*k*), according to Equation (9).

The implementation of the online LMS associated with each synaptic weight, according to Equation (9), is presented in the diagram shown in Figure 6. All *M* × *H* weights are updated in parallel and stored in the registers, RW*mh*. The RMU register stores the value of the learning rate, *μ*, and to avoid problems in realigning the algorithm, the operations of addition, subtraction and multiplication are implemented with zero delay.

### 3.5. Delays of Operations

Due to the reduction in the size of transistors and increases in clock frequency, delays caused by interconnection paths (also known as routing delays) are one of the dominant factors affecting time restrictions. In FPGAs, routing delays are caused by programmable routing switches, which significantly increases the wire delay [27]. Techniques, such as wire pipelining (or delay padding), in which flip-flops or latches are inserted between critical stages, can reduce the paths in order to achieve the necessary time restrictions [27,28].

Hence, based on the technique of wire pipelining, it can be seen from Equations (11), (18) and (19) that there are delays associated with the operations of addition, subtraction, multiplication and ROM reading in the structures presented in Figures 3, 4 and 5. The delays are implemented by the addition of flip-flops or latches after the operations, represented here by the variables *a*, *r*, *u*, *c* and *q*. It is important to highlight that the operations (addition and multiplication) in the updating circuit of the synaptic weights, *w_mh_* (*k*) (see Figure 6), do not use delays (wire pipelining technique). This restriction ensures synchronization of updating of the weights with their corresponding inputs.

It can be seen from the case studies presented in [29–33] using Virtex-6 that the value of the delay influences the maximum clock frequency associated with the arithmetic operation in question. For example, in [29], it was observed that the operation of addition in Virtex-6 for variables of 32 bits with a signal could achieve a maximum clock frequency of 410 MHz, using a delay of *z*^−^*^3^*, while a maximum clock frequency of 388 MHz was obtained in the absence of any delay.

### 3.6. Analysis of the Area Occupied

The occupied area (in FPGA) of the RBF network can be expressed as:

(20) $$\begin{array}{lll} A^{\text{RBF}}(n,a,r,c,u,p,H,M,\alpha ,\gamma ,\eta ,\kappa ,P,D_{1},D_{2}) & = & \left\{A^{\text{ILM}}\left(n,a,r,p,H,P,\gamma ,\alpha \right)\right\}\\ & & +\left\{A^{\text{OLM}}\left(n,c,u,M,H,D_{1},D_{2},\eta \right)\right\}\\ & & +\left\{A^{\text{UAM}}\left(n,M,H,\kappa ,D_{1},D_{2}\right)\right\} \end{array}$$

where:

(21) $$\begin{array}{lll} A^{\text{ILM}}\left(n,a,r,p,H,\alpha ,\gamma ,P\right) & = & \{N_{FF}^{\text{ILM}}\left(n,a,r,p,H,P,\gamma ,\alpha \right)\\ & & N_{\text{LUT}}^{\text{ILM}}\left(n,a,r,p,H,P,\gamma ,\alpha \right)\\ & & N_{\text{EmbMult}}^{\text{ILM}}\left(p,H,\gamma \right),N_{\text{BRAM}}^{\text{ILM}}\left(n,H,\alpha ,P\right)\} \end{array}$$

(22) $$\begin{array}{lll} A^{\text{OLM}}\left(n,c,u,M,H,\eta ,D_{1},D_{2}\right) & = & \{N_{FF}^{\text{OLM}}\left(n,c,u,M,H,D_{1},D_{2},\eta \right),\\ & & N_{\text{LUT}}^{\text{OLM}}\left(n,c,u,M,H,D_{1},D_{2},\eta \right)\\ & & N_{\text{EmbMult}}^{\text{OLM}}\left(M,H,\eta \right)\} \end{array}$$

and

(23) $$\begin{array}{lll} A^{\text{UAM}}\left(n,M,H,\kappa ,D_{1},D_{2},\right) & = & \{N_{FF}^{\text{UAM}}\left(n,M,H,\kappa ,D_{1},D_{2},\right),N_{\text{LUT}}^{\text{UAM}}\left(n,M,H,\kappa ,D_{1},D_{2}\right)\\ & & N_{\text{EmbMult}}^{\text{UAM}}\left(M,H,\kappa \right)\} \end{array}$$

where *A^ILM^*, *A^OLM^* and *A^UAM^* are collections formed by the number of flip-flops, *N_FF_*, number of LUTs, *n_LUT_* and number of embedded multipliers, *n_Mult_*, used by modules ILM, OLM and UAM, respectively *n_BRAM_* is the number of embedded blocks RAMs, for which ILM can be used to implement the *H* ROMs that represent the radial function [34]. It is important to observe that the ROMs can be also implemented for logic cells.

The ILM module can be expressed by:

(24) $$\begin{array}{lll} N_{FF}^{\text{ILM}}\left(n,a,r,p,H,P,\gamma ,\alpha \right) & \leq & HpN_{FF}^{\text{Sub}}\left(n,a\right)+H\left(\overset{\left\lceil \log_{2}(p)\right\rceil -1}{\underset{i=0}{\text{∑}}}2^{i}\right)N_{FF}^{\text{Add}}\left(n,a\right)\\ & & +\gamma \left(1+H\right)N_{FF}^{\text{Mult}}\left(n,r\right)+HpN_{FF}^{RC}\left(n\right)+HN_{FF}^{\text{BETA}}\left(n\right)\\ & & +\alpha N_{FF}^{\text{ROM}}\left(n,q,P\right)+\left(H-\alpha \right)N_{FF}^{ROM_{\text{BRAM}}}\left(n,q,P\right) \end{array}$$

where $N_{FF}^{\text{Sub}}\left(n,a\right)$ and $N_{FF}^{\text{Add}}\left(n,a\right)$ are the number of flip-flops required to implement a subtraction and addition of *n* bits with a delay of *a*, respectively. $N_{FF}^{\text{Mult}}\left(n,r\right)$ is the number of flip-flops required to implement a multiplication of *n* bits with a delay of *r*. $N_{FF}^{RC}\left(n\right)$ and $N_{FF}^{\text{BETA}}\left(n\right)$ represent the number of flip-flops required to implement each register RC*hp* and BETA*h*, respectively. The variable $N_{FF}^{\text{ROM}}\left(n,q,P\right)$ is the number of flip-flops used to implement with logic cells, the *h*-th ROM with depth *P* and *n* bits (see Equation (15)). $N_{FF}^{ROM_{\text{BRAM}}}\left(n,q,P\right)$ represents the situation where the ROM is implemented by block RAMs. The parameter *γ* is the portion of the *Hp* multipliers (ILM) that are implemented by logic cells, and *α* is part of the *H* ROMs, which are also implemented in logic cells. The number of LUTs can be expressed by:

(25) $$\begin{array}{lll} N_{\text{LUT}}^{\text{ILM}}\left(n,a,r,p,H,P,\gamma ,\alpha \right) & \leq & HpN_{\text{LUT}}^{\text{Sub}}\left(n,a\right)+H\left(\overset{\left\lceil \log_{2}(p)\right\rceil -1}{\underset{i=0}{\text{∑}}}2^{i}\right)N_{\text{LUT}}^{\text{Add}}\left(n,a\right)\\ & & +\gamma \left(1+H\right)N_{\text{LUT}}^{\text{Mult}}\left(n,r\right)+HpN_{\text{LUT}}^{RC}\left(n\right)+HN_{\text{LUT}}^{\text{BETA}}\left(n\right)\\ & & +\alpha N_{\text{LUT}}^{\text{ROM}}\left(n,q,P\right)+\left(H-\alpha \right)N_{\text{LUT}}^{ROM_{\text{BRAM}}}\left(n,q,P\right)\\ & & +N_{\text{LUT}}^{\text{Rout}}\left(n\right) \end{array}$$

where $N_{\text{LUT}}^{\text{Sub}}\left(n,a\right)$ and $N_{\text{LUT}}^{\text{Add}}\left(n,a\right)$ are the number of LUTs required to implement a subtraction and addition of *n* bits with a delay of *a*, respectively. $N_{\text{LUT}}^{\text{Mult}}\left(n,r\right)$ is the number of LUTs required to implement a multiplication of *n* bits with a delay of *r*. $N_{\text{LUT}}^{RC}\left(n\right)$ and $N_{\text{LUT}}^{\text{BETA}}\left(n\right)$ represent the number of LUTs required to implement each register RC*hp* and BETA*h*, respectively. The variable $N_{\text{LUT}}^{\text{ROM}}\left(n,q,P\right)$ is the number of LUTs used to implement, with logic cells, the *h*-th ROM with depth *P* and *n* bits (see Equation (15)). $N_{\text{LUT}}^{ROM_{\text{BRAM}}}\left(n,q,P\right)$ represents the situation where the ROM is implemented by block RAMs. Finally, $N_{\text{LUT}}^{\text{Rout}}\left(n\right)$ is the number of LUTs used for routing [34]. The number of embedded multipliers used can be expressed as:

(26) $$N_{\text{EmbMult}}^{\text{ILM}}\left(P,H,\gamma \right)=\left(Hp-\gamma \right)$$

and the number of blocks RAMs as

(27) $$N_{\text{BRAM}}^{\text{ILM}}\left(n,H,\alpha ,P\right)=\left(H-\alpha \right)N_{\text{BRAM}}^{\text{ROM}}\left(n,P\right)$$

where $N_{\text{BRAM}}^{\text{ROM}}\left(n,P\right)$ is the number of blocks RAMs needed to implement each *h*-th ROM with depth *P* and *n* bits (see Equation (15)).

For the OLM module:

(28) $$\begin{array}{lll} N_{FF}^{\text{OLM}}\left(n,c,u,M,H,D_{1},D_{2},\eta \right) & \leq & M\left(\overset{\left\lceil \log_{2}(H)\right\rceil -1}{\underset{i=0}{\text{∑}}}2^{i}\right)N_{FF}^{\text{Add}}\left(n,c\right)+\eta N_{FF}^{\text{Mult}}\left(n,u\right)\\ & & +HMN_{FF}^{\text{Delay}}\left(n,D_{2}-D_{1}\right) \end{array}$$

(29) $$\begin{array}{lll} N_{\text{LUT}}^{\text{OLM}}\left(n,c,u,M,H,D_{1},D_{2},\eta \right) & \leq & M\left(\overset{\left\lceil \log_{2}(H)\right\rceil -1}{\underset{i=0}{\text{∑}}}2^{i}\right)N_{\text{LUT}}^{\text{Add}}\left(n,c\right)+\eta N_{\text{LUT}}^{\text{Mult}}\left(n,u\right)\\ & & +HMN_{\text{LUT}}^{\text{Delay}}\left(n,D_{2}-D_{1}\right)+N_{\text{LUT}}^{\text{Rout}}\left(n\right) \end{array}$$

where *η* represents the portion of *HM* multipliers (in OLM) that are implemented by logic cells. The variables $N_{FF}^{\text{Delay}}\left(n,D_{2}-D_{1}\right)$ and $N_{\text{LUT}}^{\text{Delay}}\left(n,D_{2}-D_{1}\right)$ represent the number of flip-flops and LUTs required to implement a delay of size *D*_2_ − *D*_1_. The number of embedded multipliers in OLM can be expressed by:

(30) $$N_{\text{EmbMult}}^{\text{OLM}}\left(M,H,\eta \right)=\left(HM-\eta \right)$$

Finally, the expressions that estimate the occupied area of the UAM module are expressed as:

(31) $$\begin{array}{lll} N_{FF}^{\text{UAM}}\left(n,M,H,D_{1},D_{2},\kappa \right) & = & MHN_{FF}^{\text{Sub}}\left(n,0\right)+MHN_{FF}^{\text{Add}}\left(n,0\right)+\kappa N_{FF}^{\text{Mult}}\left(n,0\right)\\ & & +HMN_{FF}^{\text{Delay}}\left(n,D_{2}-D_{1}\right)+HMN_{FF}^{\text{Delay}}\left(n,D_{2}\right)\\ & & +HMN_{FF}^{RW}\left(n\right) \end{array}$$

(32) $$\begin{array}{lll} N_{\text{LUT}}^{\text{UAM}}\left(n,M,H,D_{1},D_{2},\kappa \right) & = & MHN_{\text{LUT}}^{\text{Sub}}\left(n,0\right)+MHN_{\text{LUT}}^{\text{Add}}\left(n,0\right)+\kappa N_{\text{LUT}}^{\text{Mult}}\left(n,0\right)\\ & & +HMN_{\text{LUT}}^{\text{Delay}}\left(n,D_{2}-D_{1}\right)+HMN_{\text{LUT}}^{\text{Delay}}\left(n,D_{2}\right)\\ & & +HMN_{\text{LUT}}^{RW}\left(n\right)+N_{\text{LUT}}^{\text{Rout}}\left(n\right) \end{array}$$

where *κ* represents the portion of the 2*HM* multipliers that are implemented in logic cells. $N_{FF}^{RW}\left(n\right)$ and $N_{\text{LUT}}^{RW}\left(n\right)$ represent the number of flip-flops and LUTs required to implement each register RW*mh*, respectively. The number of embedded multipliers in UAM can be expressed by:

(33) $$N_{\text{EmbMult}}^{\text{UAM}}\left(M,H,\kappa \right)=\left(2HM-\kappa \right)$$

## 4. Results and Experimental Tests

Two operational scenarios were analyzed in order to validate the implementation of the RBF in an FPGA. The first scenario was a widely known problem of nonlinear classification, in which the RBF attempted to copy the functioning of the XOR gate. In the second scenario, the RBF performed interpolation of the sine function. Tables 1–3 present the parameters used in the experimental tests involving the two scenarios. The results were obtained using the System Generator development platform (Xilinx) [23] and a Virtex-6 xc6vcx240t 1ff1156 FPGA. The Virtex-6 FPGA possesses 37,680 slices grouping 301, 440 flip-flops, 150, 720 LUTs that can be used to implement logic functions or memories and 768 DSPcells (Virtex-6 FPGA DSP48E1) with multipliers and accumulators [23,29,33]. In both scenarios, the signal sampling rate was $R_{s}=\frac{1}{T_{s}}$, where *T_s_* is the time between the *k*-th samples.

In the results described in this section, the operations of addition and subtraction were implemented with logic cells utilizing LogiCORE IP Adder/Subtracter v11.0 (Xilinx) [30]. All of the multiplication operations were implemented using embedded multipliers (*γ* = *η* = *κ* = 0) of the type Virtex-6 FPGA DSP48E1 Slice with LogiCORE IP Multiplier v11.2 [29,33], and all of the ROMs associated with the radial functions were implemented using logic cells (*α* = *H*) with LogiCORE IP Distributed Memory Generator v6.3 [32].

In the Virtex-6 FPGA, the arithmetic operations (addition, subtraction and multiplication) do not possess restrictions related to the delays (*a* ≥ 0, *r* ≥ 0, *u* ≥ 0 and *c* ≥ 0). Meanwhile, as described in Subsection 3.5, the technique of wire pipelining can assist in the routing process, reducing the distance between the operations in order to achieve the time restrictions. In the case of the ROM, implementation in the Virtex-6 FPGA requires the use of internal memory blocks (Virtex-6 block RAMs) with *q* > 0. In the absence of this condition, *q* ≥ 0 (in this case, the ROM is implemented by logic cells) [31,32]. Table 3 presents all of the delay values used in the simulations, where the values selected were based on test cases presented in [29–31], where various delay values and the maximum frequencies obtained are illustrated. For example, in [29], it was found that the multiplication operation for variables of 18 bits with a signal could achieve a maximum clock frequency of 450 MHz, using a delay of *r* = 3.

### 4.1. Results Obtained for Synthesis of the RBF in the FPGA

Tables 4 and 5 present the results obtained after the process of synthesis of the RBF (with the parameters presented in Tables 1–3) in the FPGA. For both scenarios, it can be clearly seen that the sampling rate and the area of occupation were highly sensitive to the number of bits. On the other hand, due to the parallelization, differences between the two scenarios were not large, using the same quantity of bits (14, 15 and 16).

Occupation of the area used by the registers is due to the storage of fixed centers (RC*hi*), constants (Beta*h*, RMU), synaptic weights (RW*mh*) and delays. This is affected to a greater extent by the architecture of the ANN (number of inputs, *p*, number of centers, *H*, and number of outputs, *M*) than by the number of bits. Occupation of the logic cells is related to the addition operations performed using the construction of logic functions, as well as the radial functions that were developed by means of the construction of ROM memories, as described in Section 3.2. In the latter case, the degree of precision and the architecture of the network exert a direct influence, as shown in Tables 4 and 5. The multiplication operations were synthesized in internal DSP circuits, as a result of which, the area consumed by these operations remained constant, in terms of the number of bits, and only altered in terms of structural changes in the network.

### 4.2. Precision of the Response

**Figure 7.**
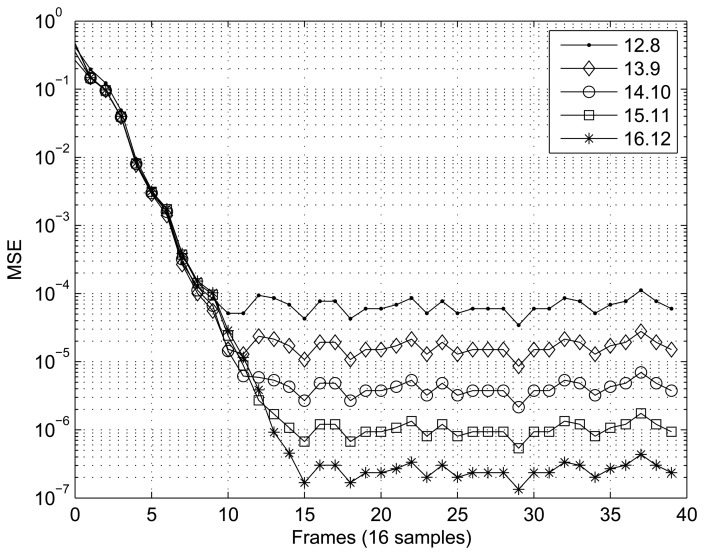
Performance of the RBF in tests using the XOR gate scenario.

**Figure 8.**
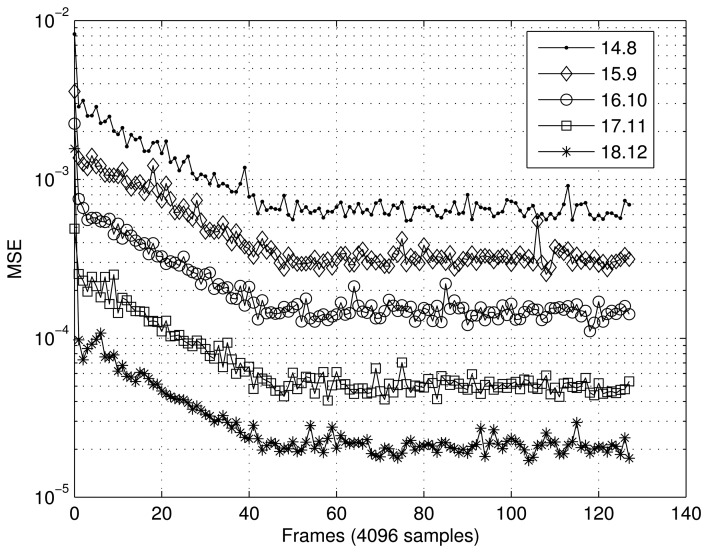
Performance of the RBF in tests using the sine function scenario.

Figures 7 and 8 present the results obtained for the MSE as a function of the number of samples in the two scenarios tested. In the case of the first scenario (XOR gate) (Figure 7), the MSE was calculated using frames of 16 samples, and 40 frames were tested. For the scenario involving interpolation of the sine function (Figure 8), frames of 4096 sample were utilized to calculate the MSE, and 128 frames were tested. In both cases, the RBF implemented showed satisfactory convergence, with the best results being directly related to the number of bits, *n*.

### 4.3. Estimate of the Area Occupied

**Figure 9.**
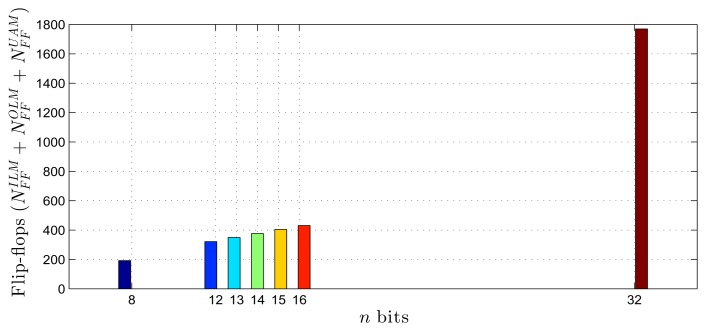
Area occupied (flip-flops) for different fixed point formats, in the XOR gate scenario.

**Figure 10.**
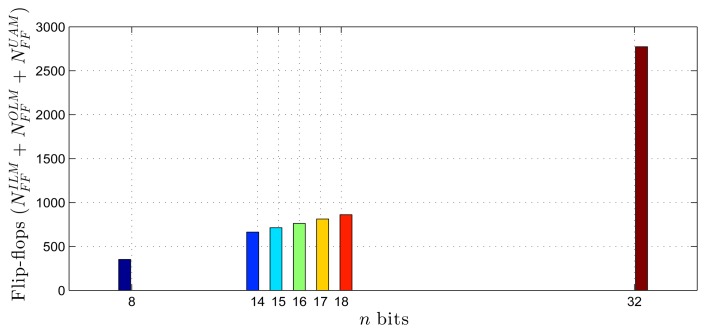
Area occupied (flip-flops) for different fixed point formats, in the sine function scenario.

**Figure 11.**
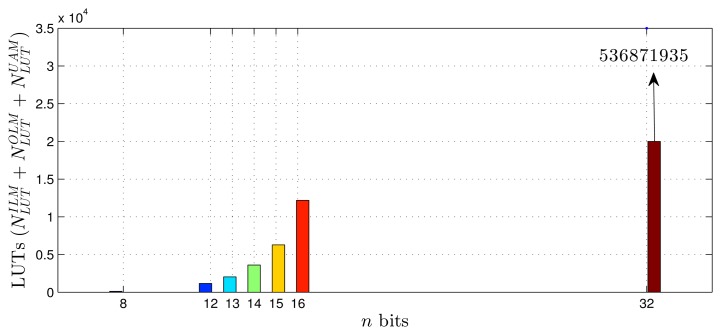
Area occupied (LUTs) for different fixed point formats, in the XOR gate scenario.

**Figure 12.**
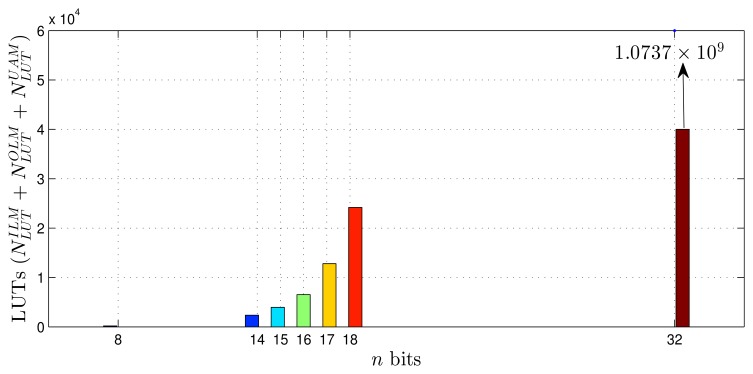
Area occupied (LUTs) for different fixed point formats, in the sine function scenario.

Using as a reference the parameters presented in Tables 1–3, Equations (20)–(27) and the estimates provided by the Xilinx manufacturer (see Tables 6 and 7) [29–33], it was possible to obtain estimated area occupation limits for the flip-flops and LUTs with *n* = 8 and *n* = 32 bits, as shown in Figures 9, 10, 11 and 12. These figures also show the real results obtained after the synthesis in the case of the XOR gate (*n* = {12, … , 16}) and the sine function (*n* = {14, … , 18}). Since embedded (fixed) multipliers were used, the multiplications did not influence the area occupied. However, it is important to point out that if the onboard multipliers are not sufficient, multipliers could be constructed with logic cells. It is important to observe that the number of bits will result in an exponential growth in the occupied area.

**Figure 13.**
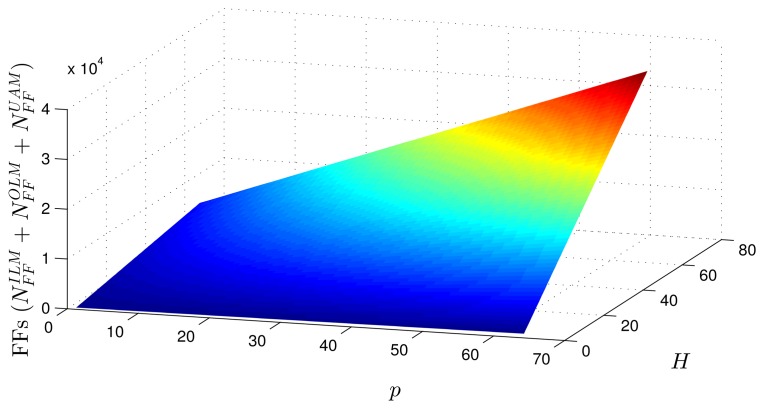
Area occupied (flip-flops) for different numbers of inputs, *p*, centers, *H* and *M* = 1.

**Figure 14.**
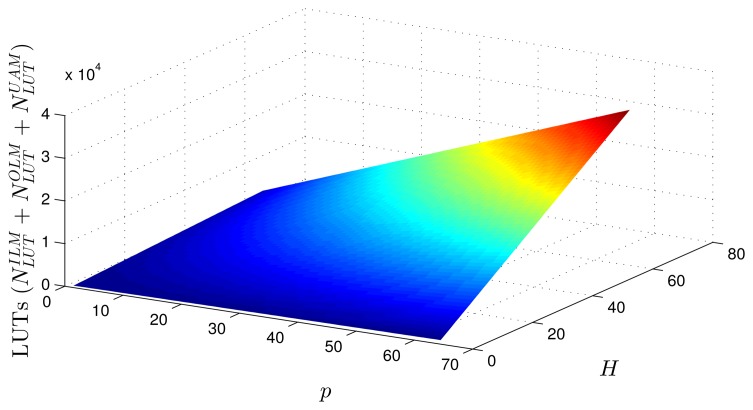
Area occupied (LUTs) for different numbers of inputs, *p*, centers, *H* and *M* = 1.

Figures 13 and 14 show occupation estimates for various numbers of inputs, *p*, and centers, *H*, for *M* = 1 and *n* = 8. The curves demonstrate that the occupation capacity is more influenced by the number of bits, *n* (exponential increase), than by the structure of the RBF that grows linearly with the parameters *p*, *H* and *M*. The curves illustrated in Figures 13 and 14 show that, for example, with eight bits, it is possible to work with a relatively large RBF (*p* = 64, *H* = 64 and *M* = 1) using around 50% occupancy of a Virtex 6 (40, 000 LUTs and flip-flops more multipliers). This RBF configuration could be used in adaptive equalizers [35,36] and in some applications in mobile robotics [37].

### 4.4. Real-World Cases

Artificial neural networks of the type RBF can be applied to different real-world problems that may or may not require online training. Amongst these problems can be highlighted the use of RBF networks in adaptive equalizers for wireless communication systems [35,36], for obstacle avoidance in mobile robotics [37], in pattern recognition [38], as well as other applications. Nevertheless, for problems that require online training, the speed of the training in terms of the number of iterations per second is a fundamental determinant of viability in dedicated hardware. The development of the present work is based on this perspective.

Analysis of the results obtained for time (or, in other words, the sampling rate, *R*, of the ANN) showed that in both cases (XOR gate and sine function), the value was much more closely associated with the number of bits, *n*, than with the structure of the network, which was due to the parallel implementation proposed in this paper. From the results obtained, it could be seen that if space exists in the FPGA for the implementation of the ANN, the sampling rate essentially depends on the number of bits. For example, in the case of the FPGA used here, it was possible to achieve sampling rates of around 100 MHz (see Tables 4 and 5); in other words, to work at 100 mega-iterations per second.

## 5. Conclusions

This work presents a parallel fixed point implementation, in an FPGA, of an RBF trained with an online LMS algorithm. The implementation of the RBF was analyzed in terms of occupation area, bit resolution and processing delay. The proposed structure was tested at different resolutions, using two widely known scenarios, namely the problem of nonlinear classification with the XOR gate and the problem of interpolation utilizing the sine function. The results obtained were highly satisfactory, indicating the potential feasibility of the technique for use in practical situations of greater complexity.

## Figures and Tables

**Table 1.** Parameters used for the XOR gate scenario.

Number of inputs (*p*)	1
Number of centers (*H*)	2
Number of neurons of the output layer (*M*)	1
Scaling parameter associated radial function (*β*)	2
Learning rate (*μ*)	0.3125

**Table 2.** Parameters used for the sine function interpolation scenario.

Number of inputs (*p*)	1
Number of centers (*H*)	4
Number of neurons of the output layer (*M*)	1
Scaling parameter associated radial function (*β*)	0.125
Learning rate (*μ*)	0.5

**Table 3.** Delays associated with the arithmetical operations used in the two scenarios.

*z*^−^*^r^*	3
*z*^−^*^a^*	0
*z*^−^*^q^*	1
*z*^−^*^u^*	0
*z*^−^*^c^*	0

**Table 4.** Processing speed and area occupied for different fixed point formats, in the XOR gate scenario. LUT, lookup table.

**Fixed Point Format ([*n*.*b*])**	**Sample Rate *R_s_* (MHz)**	**Flip-Flops and Latches**	**Logic Cells (LUTs)**	**Multipliers**
[12.8]	100.9285	321	1, 170	10
[13.9]	84.7673	349	2, 038	10
[14.10]	75.5401	376	3, 610	10
[15.11]	74.9569	404	6, 284	10
[16.12]	66.8717	431	12, 184	10

**Table 5.** Processing speed and area occupied for different fixed point formats, in the sine function scenario.

**Fixed Point Format ([*n*.*b*])**	**Sample Rate *R_s_* (MHz)**	**Flip-Flops and Latches**	**Logic Cells (LUTs)**	**Multipliers**
[14.8]	103.4554	106	2, 370	16
[15.9]	80.5477	115	3, 944	16
[16.10]	75.5589	124	6, 519	16
[17.11]	68.4650	133	12, 801	16
[18.12]	65.4332	142	24, 176	16

**Table 6.** Estimated number of flip-flops provided by the Xilinx manufacturer [29–33].

***n***	$N_{FF}^{\text{Sub}}\left(n,0\right)$	$N_{FF}^{\text{Add}}\left(n,0\right)$	$N_{FF}^{\text{Mult}}\left(n,3\right)$	$N_{FF}^{RC}\left(n\right)$	$N_{FF}^{\text{BETA}}\left(n\right)$	$N_{FF}^{RW}\left(n\right)$

8	0	0	110	8	8	8
32	91	91	–	32	32	32

***n***	$N_{FF}^{\text{Delay}}\left(n,D_{2}-D_{1}\right)$	$N_{FF}^{\text{Delay}}\left(n,D_{2}\right)$	$N_{FF}^{\text{ROM}}\left(n,1,P\right)$	$N_{FF}^{\text{Mult}}\left(n,0\right)$	$N_{\text{LUT}}^{\text{Rout}}\left(n\right)$	

8	(*D*_2_ − *D*_1_) × 8	(*D*_2_) × 8	8	110	0	
32	(*D*_2_ − *D*_1_) × 32	(*D*_2_) × 32	32	–	0	

**Table 7.** Estimated number of LUTs provided by the Xilinx manufacturer [29–33].

***n***	$N_{\text{LUT}}^{\text{Sub}}\left(n,0\right)$	$N_{\text{LUT}}^{\text{Add}}\left(n,0\right)$	$N_{\text{LUT}}^{\text{Mult}}\left(n,3\right)$	$N_{\text{LUT}}^{RC}\left(n\right)$	$N_{\text{LUT}}^{\text{BETA}}\left(n\right)$	$N_{\text{LUT}}^{RW}\left(n\right)$

8	8	8	116	0	0	0
32	93	93	–	0	0	0

***n***	$N_{\text{LUT}}^{\text{Delay}}\left(n,D_{1}-D_{2}\right)$	$N_{\text{LUT}}^{\text{Delay}}\left(n,D_{2}\right)$	$N_{\text{LUT}}^{\text{ROM}}\left(n,1,P\right)$	$N_{\text{LUT}}^{\text{Mult}}\left(n,0\right)$	$N_{\text{LUT}}^{\text{Rout}}\left(n\right)$	

8	0	0	≈ 2^(8−4)^	116	0	
32	0	0	≈ 2^(32−4)^	–	0	
